# Continued Evolution of West Nile Virus, Houston, Texas, USA, 2002–2012

**DOI:** 10.3201/eid1909.130377

**Published:** 2013-09

**Authors:** Brian R. Mann, Allison R. McMullen, Daniele M. Swetnam, Vence Salvato, Martin Reyna, Hilda Guzman, Rudy Bueno, James A. Dennett, Robert B. Tesh, Alan D.T. Barrett

**Affiliations:** University of Texas Medical Branch, Galveston, Texas, USA (B.R. Mann, A.R. McMullen, D.M. Swetnam, H. Guzman, R.B. Tesh, A.D.T. Barrett);; Harris County Public Health and Environmental Services, Houston, Texas, USA (V. Salvato, M. Reyna, R. Bueno, Jr., J.A. Dennett)

**Keywords:** West Nile virus, WNV, flavivirus, viruses, virus surveillance, viral epidemiology, virus evolution, phylogenetics, Texas

## Abstract

We investigated the genetics and evolution of West Nile virus (WNV) since initial detection in the United States in 1999 on the basis of continual surveillance studies in the Houston, Texas, USA, metropolitan area (Harris County) as a surrogate model for WNV evolution on a national scale. Full-length genomic sequencing of 14 novel 2010–2012 WNV isolates collected from resident birds in Harris County demonstrates emergence of 4 independent genetic groups distinct from historical strains circulating in the greater Houston region since 2002. Phylogenetic and geospatial analyses of the 2012 WNV isolates indicate closer genetic relationship with 2003–2006 Harris County isolates than more recent 2007–2011 isolates. Inferred monophyletic relationships of these groups with several 2006–2009 northeastern US isolates supports potential introduction of a novel WNV strain in Texas since 2010. These results emphasize the need to maintain WNV surveillance activities to better understand WNV transmission dynamics in the United States.

The emergence of West Nile virus (WNV) in the Western Hemisphere in 1999 poses an ongoing public health threat in North America as the most common cause of epidemic encephalitis in the United States (*1*). WNV transmission is maintained in an enzootic cycle between mosquitoes and birds; equids, humans, other mammals, and some bird species act as dead-end hosts (*2*). Human infections are asymptomatic in 80% of cases, and West Nile fever develops in ≈20% of infected patients, which progresses to neuroinvasive disease in <1% (*3*).

After introduction of WNV in the United States in 1999 (*4*), local transmission of the original New York genotype (NY99) in resident *Culex* spp. mosquito and wild bird populations fueled the geographic expansion of WNV from the northeastern region across the continental United States, north into Canada, and south into Central and South America (*5**–**7*). Subsequent introduction into Texas in 2002 resulted in 105 confirmed human infections, high mortality rates among local corvids, and a 31.2% seroconversion rate among resident birds of Harris County, Texas (Houston metropolitan area) alone (*8*). Uninterrupted surveillance of the related St. Louis encephalitis virus in local *Culex* spp. mosquito populations by the Harris County Mosquito Control Division since 1964 provided an ideal infrastructure for the expanded detection of WNV activity in the mosquito vector and the wild bird reservoir on a major bird migratory pathway. Routine collections of WNV-positive birds and mosquito pools to date have provided an outstanding opportunity to investigate WNV diversity and evolution on a fine geographic scale comparable to similar surveillance foci in the midwestern and New England regions of the United States (*9**–**11*). However, because of its geographic location, Harris County represents a different ecosystem, namely a warm year-round climate with unique resident mosquito and avian species.

Phylogenetic examination of 2002–2004 Harris County isolates confirmed rapid displacement of the NY99 genotype with the novel North American genotype (NA/WN02) in 2002 (*12*). Fine-scale geospatial genetic comparisons of these isolates provided further evidence of increased WNV genetic diversification in the greater Houston region relative to the homogenous distribution of the now extinct NY99 genotype (*12*,*13*). Subsequently, McMullen et al. identified the emergence of the southwestern genotype (SW/WN03) in the southwestern United States in 2003 and positive selection for the encoded NS4A-A85T and NS5-K314R amino acid substitutions in the WNV nonstructural (NS) proteins (*14*). To date, the NA/WN02 and SW/WN03 genotypes still appear to co-circulate.

Endemic transmission of WNV in the United States since 2006 has shown a dramatic decrease in the confirmed incidence of clinical WNV disease; <1,100 annual human cases were reported during 2008–2011 (*15*). Despite identification of regional heterogeneous WNV populations, a relative stasis in WNV evolution has been observed in Harris County, consistent with the logistic molecular clock model and a decreasing viral growth rate proposed on a national scale (*16**–**18*). Notably, the current 2012 WNV transmission season demonstrates major divergence from this status quo; >5,600 human infections have been reported nationwide (*15*). Incidence of clinical WNV disease in the Texas outbreak alone accounted for >33% of the cases in the United States (1,868 cases, including 844 reports of neuroinvasive disease and 89 deaths) and >994 confirmed cases in the greater Dallas/Fort Worth, Texas, metropolitan area and 101 cases in Harris County (*15*,*19*). These changes reflect final US and Texas and WNV cases for 2012 reported by the Centers for Disease Control and Prevention (Atlanta, GA, USA) (www.cdc.gov/media/releases/2013/a0513-west-nile.html) after submission of this report after review. Therefore, studies concerning the continued evolution of WNV in the central and southern United States remain vital for elucidating the role of dynamic genetic heterogeneity and accumulation of novel mutations in the transmission dynamics and incidence of clinical WNV disease.

We report consensus sequence analyses of 17 novel full-length 2010 (n = 1), 2011 (n = 1), and 2012 (n = 15) WNV isolates collected from WNV-positive birds and *Culex* spp. mosquito pools in Harris County Texas (n = 14) and the greater Dallas/Fort Worth region (n = 3). Inclusion of these new isolates with 28 additional 2002–2009 Harris County WNV isolates in phylogenetic and geospatial analyses of the greater Houston region provides an ideal model for investigating the role of ongoing WNV evolution relative to environmental and clinical incidence reported over the past decade. Furthermore, isolates from the recent WNV epidemic demonstrate closer phylogenetic relationships with original 2002–2003 Harris County isolates, inconsistent with phylogenetic trends observed until 2011 and supporting evidence for the recent introduction of a novel WNV strain(s) in Texas from another geographic region.

## Materials and Methods

### Virus Isolates

The World Reference Center for Emerging Viruses and Arboviruses at the University of Texas Medical Branch in Galveston, Texas, supplied the WNV isolates characterized herein provided by the Harris County Mosquito Control Division and Texas Department of State Health Services (Table 1). The fourteen 2010–2012 Harris County isolates were cultured from brain tissue of collected dead WNV-positive birds in Vero cells at the University of Texas Medical Branch in Galveston. Secondary passage of each isolate in Vero cells provided working stocks that were stored at –80°C. These same procedures were used for the 3 Dallas/Fort Worth mosquito isolates.

**Table 1:** West Nile virus isolates described in sequence and phylogenetic analyses, Harris County , Texas, USA,1998–2012*

Strain	Map code†	Location	Zip code‡	Source§	Collection year	GenBank accession no.
IS-98 STD	–	Eilat, Israel	NA	White stork	1998	AF481864
NY99-flamingo382-99	–	New York, NY, USA	NA	Chilean flamingo	1999	AF196835
Kuritz [TVP 8553]	–	Beaumont, TX, USA	NA	Human	2002	AY289214
TX114	B1-1	Harris Co., TX, USA	77043	Blue jay	2002	GU827998
TX 2002 1	–	Harris Co., TX, USA	NA	Human	2002	DQ164198
TX 2002 2	–	Harris Co., TX, USA	NA	Human	2002	DQ164205
TX1153	B2-1	Harris Co., TX, USA	77077	Mourning dove	2003	AY712945
TX1171	B3-1	Harris Co., TX, USA	77030	Blue jay	2003	AY712946
TX1175	B4-3	Harris Co., TX, USA	77346	Blue jay	2003	GU828000
TX1461	–	Harris Co., TX, USA	NA	Avian	2003	AY712947
TX 2003	–	Harris Co., TX, USA	NA	Human	2003	DQ164199
v4095	M10-2	Harris Co., TX, USA	77093	*Culex quinquefasciatus*	2003	GU828002
v4369	M11-2	Harris Co., TX, USA	77039	*Cx. quinquefasciatus*	2003	AY712948
v4380	M12-2	Harris Co., TX, USA	77093	*Cx. quinquefasciatus*	2003	GU828001
M12214	M1-5	Harris Co., TX, USA	77020	*Cx. quinquefasciatus*	2005	JF415914
TX5058	B5	Harris Co., TX, USA	77057	Blue jay	2005	JF415929
M6019	M2-6	Harris Co., TX, USA	77026	*Cx. quinquefasciatus*	2006	JF415930
TX5810	B6-6	Harris Co., TX, USA	77345	Common grackle	2006	JF415915
TX6276	B7-10	Harris Co., TX, USA	77373	Northern mockingbird	2006	JF415916
M19433	M3-5	Harris Co., TX, USA	77020	*Cx. quinquefasciatus*	2007	JF415919
TX6647	B8-5	Harris Co., TX, USA	77084	Blue jay	2007	JF415917
TX6747	B9	Harris Co., TX, USA	77346	Blue jay	2007	JF415918
TX7191	B10	Harris Co., TX, USA	77005	Blue jay	2007	JF415920
TX7558	B11-5	Harris Co., TX, USA	77375	Blue jay	2008	JF415921
M20122	M4-4	Harris Co., TX, USA	77026	*Aedes albopictus*	2009	JF415928
M20140	M5-4	Harris Co., TX, USA	77021	*Ae. albopictus*	2009	JF415926
M20141	M6-4	Harris Co., TX, USA	77021	*Cx. quinquefasciatus*	2009	JF415927
M37012	M7-4	Harris Co., TX, USA	77021	*Cx. quinquefasciatus*	2009	JF415922
M37906	M8-4	Harris Co., TX, USA	77021	*Cx. quinquefasciatus*	2009	JF415923
M38488	M9-4	Harris Co., TX, USA	77004	*Ae. albopictus*	2009	JF415925
TX7827	B12-7	Harris Co., TX, USA	77060	Blue jay	2009	JF415924
**TX8092**	**B13-7**	**Harris Co., TX, USA**	**77084**	**House sparrow**	**2010**	**KC333374**
**TX8349**	**B14-5**	**Harris Co., TX, USA**	**77016**	**House sparrow**	**2011**	**KC333375**
**TX8546**	**B15-9**	**Harris Co., TX, USA**	**77065**	**Blue jay**	**2012**	**KC333376**
**TX8551**	**B16-10**	**Harris Co., TX, USA**	**77449**	**Blue jay**	**2012**	**KC333377**
**TX8559**	**B17-9**	**Harris Co., TX, USA**	**77506**	**Blue jay**	**2012**	**KC333378**
**TX8560**	**B18-8**	**Harris Co., TX, USA**	**77062**	**Blue jay**	**2012**	**KC333379**
**TX8562**	**B19-10**	**Harris Co., TX, USA**	**77450**	**Blue jay**	**2012**	**KC333380**
**TX8567**	**B20-9**	**Harris Co., TX, USA**	**77065**	**Blue jay**	**2012**	**KC333381**
**TX8571**	**B21-8**	**Harris Co., TX, USA**	**77059**	**Blue Jay**	**2012**	**KC333382**
**TX8572**	**B22**	**Harris Co., TX, USA**	**77080**	**Blue jay**	**2012**	**KC333383**
**TX8589**	**B23-10**	**Harris Co., TX, USA**	**77049**	**Loggerhead shrike**	**2012**	**KC333384**
**TX8590**	**B24-10**	**Harris Co., TX, USA**	**77339**	**Blue jay**	**2012**	**KC333385**
**TX8599**	**B25-8**	**Harris Co., TX, USA**	**77058**	**Blue jay**	**2012**	**KC333386**
**TX8604**	**B26**	**Harris Co., TX, USA**	**77021**	**House sparrow**	**2012**	**KC333387**
**TX AR12-1486**	**–**	**Denton Co., TX, USA**	**NA**	***Cx. quinquefasciatus***	**2012**	**KC711057**
**TX AR12-1648**	**–**	**Collin Co., TX, USA**	**NA**	***Cx. quinquefasciatus***	**2012**	**KC711058**
**TX AR12-10674**	**–**	**Collin Co., TX, USA**	**NA**	***Cx. restuans***	**2012**	**KC711059**

**Figure 1:**
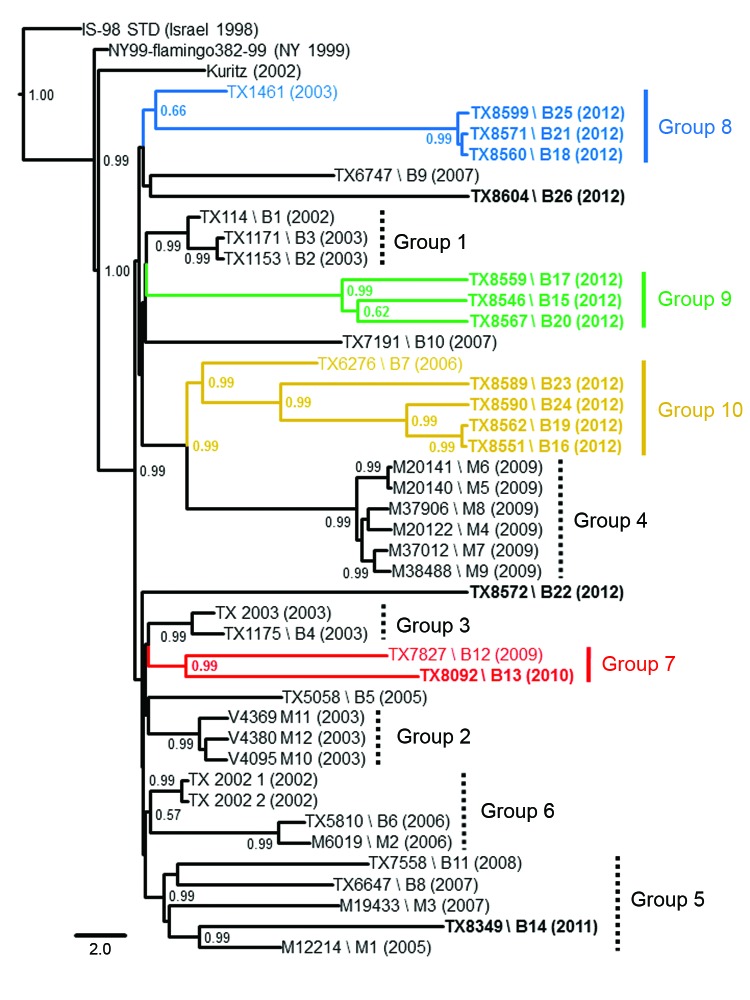
Bayesian-inferred, 50% majority-rule, coalescent phylogenetic tree of published, full-length West Nile virus isolates, Harris County, Texas, USA, 2002–2012. Novel 2010–2012 Harris County isolates cluster into 4 distinct monophyletic groups designated group 7 (red), group 8 (blue), group 9 (green), and group 10 (yellow). Strain names link geographic map code (e.g., B1, B2, M1, M2) with year of collection annotated in parentheses. Isolates sequenced in this study are indicated in **boldface**. Posterior probabilities ≥0.90 are indicated along branches to provide statistical support for inferred topologies. Scale bar indicates divergence time in years.

*Strains sequenced in this study are indicated in **boldface**. Co., county. †Map code designations include B (bird) and M (mosquito) for isolates collected in Harris County, TX, with phylogenetic relationships (groups 1–10) indicated after the hyphen (see Figure 1). –, isolates collected outside Harris County, Texas, that have no known geographic collection information.  ‡Zip codes indicated for region of isolate collection within Harris County, TX (see Figure 1). NA, not available. §Mosquito (*Culex* spp.) isolates were collected from mosquito pools.

### Genomic Sequencing

Extraction of viral RNA, reverse transcription PCR, and sequencing was conducted according to established protocols (*12*,*14*). Resulting sequences were aligned and edited relative to the prototype NY99-flamingo382-99 strain (AF196835) by using ContigExpress in the VectorNTI program (Invitrogen, Carlsbad, CA, USA) (*4*). Sequences were assembled in BioEdit version 7.0.9.0 (*20*) with 358 full-length North American WNV isolates published in GenBank (as of November 2012) and the IS-98 STD (AF481864) Israeli isolate (*21*). The encoded 10,299 nt open reading frames (ORFs) for all 372 WNV isolates were aligned by using the MUSCLE algorithm (*22*); the ORF was used because some viruses published in GenBank include the ORF alone and not the entire genome. Two additional alignments included the ORF of all 42 2002–2012 Harris County WNV isolates with and without the three 2012 Dallas/Fort Worth isolates and the prototype NY99-flamingo382-99 and IS-98 STD reference strains. Screening for potential site-specific positive selection was performed by using the Datamonkey server (*23*,*24*) and the single-likelihood ancestor counting, fixed effect likelihood (FEL), and internal branches FEL methods (*25*,*26*). Positive selection was defined as d_N_>d_S_ and a p value <0.05 in >1 method.

### Phylogenetic Analyses

Neighbor-joining (NJ) phylogenetic analyses of all alignments were processed in Seaview version 4.3.0 by using the Hasegawa–Kishino–Yano 85 substitution model and 10,000 bootstrap replicates (*27*). Maximum-likelihood (ML) analyses used RAxML-HPC Blackbox version 7.3.2 (*28*,*29*) on the CIPRES Science Gateway version 3.1 server (*30*) with the generalized time reversible substitution model with invariable sites, a gamma distribution, and 1,000 bootstrap replicates. Bayesian-inferred coalescent phylogenies were produced in BEAST version 1.6.2 (http://beast.bio.ed.ac.uk) by using the generalized time reversible substitution model with invariable sites and a gamma distribution with applied taxa dates, an uncorrelated lognormal relaxed clock model, and Bayesian Skyline prior constraints (*31*). Resulting BEAST.log and TRE files were down-sampled from triplicate 50,000,000 state runs by using LogCombiner version 1.7.4 and validation in Tracer version 1.5 (*31*). Inferred phylogenetic trees were edited in FigTree version 1.3.1 (www.mybiosoftware.com/phylogenetic-analysis/2407). In all NJ and ML tree topologies, the IS-98 STD isolate was used as a common phylogenetic outgroup.

### Statistical Analyses

The Fischer exact test and post hoc analyses were performed at α = 0.05 (IBM SPSS statistics version 20; IBM, Armonk, NY, USA) to test the association between year of collection and determined phylogenetic groupings. Adjusted standardized residuals (z-scores) at α = 0.05 were compared against the critical z-value (± 1.96) with a Bonferroni correction for multiple comparisons.

## Results

### WNV Collection

A total of 14 WNV isolates from Harris County (Table 1) were examined. Two isolates were collected from house sparrows (*Passer domesticus*) in 2010 and 2011, and 12 isolates were obtained from WNV-positive birds in 2012: 10 from blue jays (*Cyanocitta cristata*) and 1 each from a loggerhead shrike (*Lanius ludovicianus*) and a house sparrow.

Phylogenetic and geospatial analyses characterized herein compared all 14 WNV isolates with 28 other published Harris County isolates collected during 2002–2009 from mosquito pools (n = 12), birds (n = 13), or humans (n = 3) (*12*,*14*,*16*). Genomic sequences were determined for WNV isolates from each year except in 2004: 2002 (n = 3), 2003 (n = 8), 2005 (n *=* 2), 2006 (n = 3), 2007 (n = 4), 2008 (n *=* 1), and 2009 (n = 7). Sample coverage was restricted on the basis of sequence and sample availability. The Kuritz (also known as TVP8533; GenBank accession no. AY289214) isolate (Southeast Coastal Texas genotype) was included as a common Texas outgroup, and the TX114 (Bird114; GU827998) isolate served as the prototypic member of the NA/WN02 genotype (*12*,*32*).

### Divergent WNV Evolution

#### Nucleotide Changes

Comparison of the 14 novel Harris County isolates with the NY99 (NY99-flamingo382-99) prototype strain identified 45–79 nt differences (0.41%–0.72%) per 11,029-nt genome (*4*). Each isolate encodes 8 of the 13 nt changes characteristic of the NA/WN02 genotype (*12*). In addition, novel U→C transitions at positions 7015 in the NS4B gene and 8811 in the NS5 gene were conserved in 11 of the 12 Harris County isolates from 2012 (except TX8604).

#### Amino Acid Substitutions

These 14 isolates differed at 48 unique residues in the encoded 3,433-aa polyprotein relative to NY99 and had 2–10 (0.06%–0.29%) substitutions per isolate. Each isolate encoded the E-V159A substitution characteristic of the NA/WN02 genotype (*12*); however, the signature NS4A-A85T SW/WN03 genotype substitution (*14*) was identified only in the 2011 TX8349 isolate. Furthermore, 11 of the 48 deduced amino acid substitutions were conserved in >1 isolate. Single-likelihood ancestor counting, FEL, and internal branches FEL method analysis identified potential positive selection of the NS2A-H119Y substitution in the 2012 TX8546 isolate.

#### Phylogenetic and Geospatial Analysis

Phylogenetic reconstruction of ancestral topologies among all 42 published 2002–2012 Harris County WNV isolates used NJ, ML, and relaxed clock Bayesian coalescent methods. Consistent tree topologies rooted to the common IS-98 STD isolate outgroup were produced with all 3 methods. Inclusion of 2010–2012 isolates retained the phylogenetic clustering of the 2002–2009 Harris County isolates within the 6 monophyletic groups (groups 1-6) proposed by McMullen et al. (*14*) (Figure 1). Conserved nucleotide and amino acid divergence among the fourteen 2010–2012 isolates indicates emergence of 4 novel monophyletic clusters of avian isolates (groups 7–10) (Table 2). Furthermore, groups 7–10 demonstrate a significant (p≤0.044) relationship between year of collection and identified monophyletic lineage with more 2012 isolates clustering within groups 8–10.

**Table 2:** Conserved amino acid substitutions in West Nile virus isolates, Harris County, Texas, USA, 2002–2012*

G	Strain†	Year	Gene

C	prM	E		NS1	NS2A				NS2B	NS3		NS4A	NS4B			NS5	
104	156	**159**	460	236	52	90	95	188	120	180	334	**85**	14	24	240	49	**314**
NY99	1999	K	V	**V**	I	I	T	M	L	R	V	E	S	**A**	S	K	I	V	**K**
1	TX114	2002	·	·	**A**	·	·	·	·	·	·	·	·	·	**·**	·	·	·	·	**·**
	TX1153	2003	·	I	**A**	·	·	·	·	·	·	·	·	·	**·**	·	·	·	·	**·**
2	v4369	2003	·	·	**A**	·	·	·	·	·	·	·	·	·	**·**	·	·	·	·	**·**
3	TX1175	2003	·	·	**A**	·	·	·	·	·	·	·	·	·	**·**	·	·	·	·	**·**
4	M37906	2009	·	·	**A**	L	·	·	·	·	·	·	·	·	**·**	·	·	M	·	**·**
	M38488	2009	·	·	**A**	L	·	·	·	·	·	·	·	·	**·**	·	·	M	·	**·**
	M20140	2009	·	·	**A**	L	·	·	·	·	·	·	·	·	**·**	·	·	M	·	**·**
5	TX7558	2008	·	·	**A**	·	·	·	·	·	·	·	·	·	**T**	·	·	·	·	**·**
	TX6647	2007	·	·	**A**	·	·	·	V	·	·	·	·	·	**T**	·	·	·	·	**·**
	TX8349	2011	·	·	**A**	·	·	·	·	F	·	·	·	·	**T**	·	·	·	·	**·**
	M19433	2007	·	·	**A**	·	·	·	·	·	·	·	·	·	**T**	·	·	M	·	**R**
6	TX 2002 1	2002	·	·	**A**	·	·	·	·	·	·	·	·	·	**·**	·	·	M	·	**·**
	TX5810	2006	·	·	**A**	·	·	·	·	·	·	·	·	·	**·**	·	·	M	·	**·**
7	TX7827	2009	·	I	**A**	·	·	·	V	·	·	·	·	·	**·**	·	·	·	·	**·**
	TX8092	2010	·	·	**A**	·	·	·	V	·	·	·	·	·	**·**	·	·	·	·	**·**
8	TX1461	2003	·	·	**A**	·	·	·	·	·	·	·	D	·	**·**	·	·	·	·	**·**
	TX8599	2012	·	·	**A**	·	·	·	·	·	·	·	D	·	**·**	·	·	·	·	**·**
9	TX8559	2012	·	·	**A**	·	·	I	·	F	·	·	·	T	**·**	I	·	·	·	**·**
10	TX6276	2006	·	·	**A**	M	·	·	·	·	·	·	·	·	**·**	·	·	M	·	**·**
	TX8589	2012	·	·	**A**	·	·	·	·	·	K	·	·	·	**·**	·	·	M	·	**·**
	TX8590	2012	R	·	**A**	·	V	·	·	·	K	I	·	·	**·**	·	·	·	I	**·**
	TX8551	2012	R	·	**A**	·	V	·	·	·	K	I	·	·	**·**	·	R	·	I	**·**

*G, group; C, capsid; prM, premembrane; E, envelope; NS, nonstructural. Amino acid changes are relative to the prototype NY99 strain AF196836. Substitutions characteristic of defined NA/WN02 and SW/WN03 genotypes are indicated in **boldface**. Dots indicate no change from the NY99 isolate. †Indicated substitutions conserved in >1 representative isolate for each of the proposed phylogenetic groups (see Figure 1).

Group 7 (0.57% nt divergence from NY99) includes the 2009 TX7827 and 2010 TX8092 isolates. Group 8 consists of the 2003 TX1461 (Bird1461) outgroup and three 2012 isolates: TX8560, TX8571, and TX8599 (0.01%–0.04%). Group 9 (0.40%–0.45%) includes three 2012 isolates: TX8546, TX8559, and TX8567. Group 10 includes the 2006 TX6276 isolate and 2012 isolates from this study: TX8551, TX8562, TX8589, and TX8590 (0.00%–0.61%). Overall, the 37 Harris County isolates in groups 1–10 differ at 80 nt positions and 27 had substitutions shared in >1 isolate (Technical Appendix). Five substitutions (prM-V156I, NS2A-M90V, NS2A-L95F, NS4B-I240M, and NS4B-E249G) were conserved in >1 phylogenetic group. Furthermore, the signature NS4A-A85T SW/WN03 genotype substitution was present only in the 2011 TX8349 and other group 5 isolates. The TX8572 and TX8604 isolates cluster as outliers (>0.44% nt divergence) to the proposed phylogenetic groups and show increased divergence (1.13% nt and 0.38% aa) between these 2 isolates.

**Figure 2:**
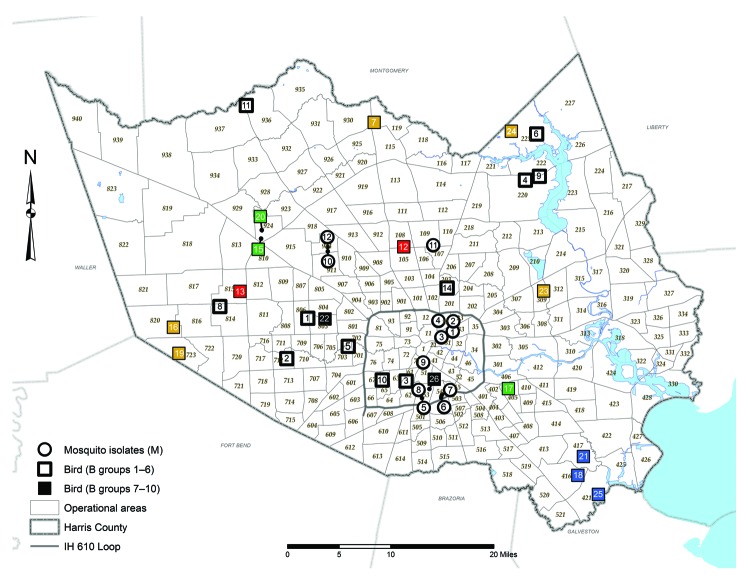
Incidence of vector-borne West Nile Virus (WNV) in Harris County, Texas, USA, 2002–2012, showing cumulative distribution of confirmed avian and mosquito (*Culex* and *Aedes* spp.) WNV isolates. Small numbers indicate reference codes for each of the 268 mosquito control operational areas. Black open symbols indicate 2002–2009 group 1–6 mosquito (circles) and bird (squares) isolates collected in mosquito control operational areas. Colored solid symbols indicate 2002–2012 Harris County isolates that cluster within monophyletic group 7 (red), group 8 (blue), group 9 (green), or group 10 (gold). Black solid symbols indicate nonclustering 2012 TX8572 and TX8604 isolates within groups 1–10. Not shown are TX 2002 1, TX 2002 2, TX 2003, and TX1461. IH, interstate highway.

Superimposition of 38 of the 42 Harris County isolates (4 isolates were excluded because of unknown collection location) on a vector-borne WNV incidence map of Harris County, based on known collection information in the 268 mosquito control operational areas, highlights the shift in WNV circulation patterns across the greater Houston region over the past decade (Figure 2). Mosquito pool isolates demonstrate robust genetic homogeneity and limited geographic distribution (Figure 2) in 9 of the 268 operational areas, suggestive of WNV overseasoning in resident mosquito populations. In particular, isolates M1 (2005) and M3 (2007) cluster within group 5, indicating transmission of the same virus strain in Houston across multiple years. In contrast, avian-derived isolates illustrate widespread incidence of similar genetic signatures in 2002–2011 WNV isolates, as highlighted in dispersal of several 2005–2011 group 5 SW/WN03 genotype isolates. However, group 7–10 isolates demonstrate comparable geographic distribution but limited monophyletic support with group 1–6 isolates. Furthermore, group 8 isolates remain geographically restricted compared to more pervasive group 10 isolates. Overall, phylogenetic and geospatial analysis of novel 2012 WNV isolates indicate a closer genetic relationship with 2003–2006 Harris County isolates than with more recent 2007–2011 WNV isolates.

### Novel Introduction Event in 2012 WNV Outbreak in Texas

**Figure 3:**
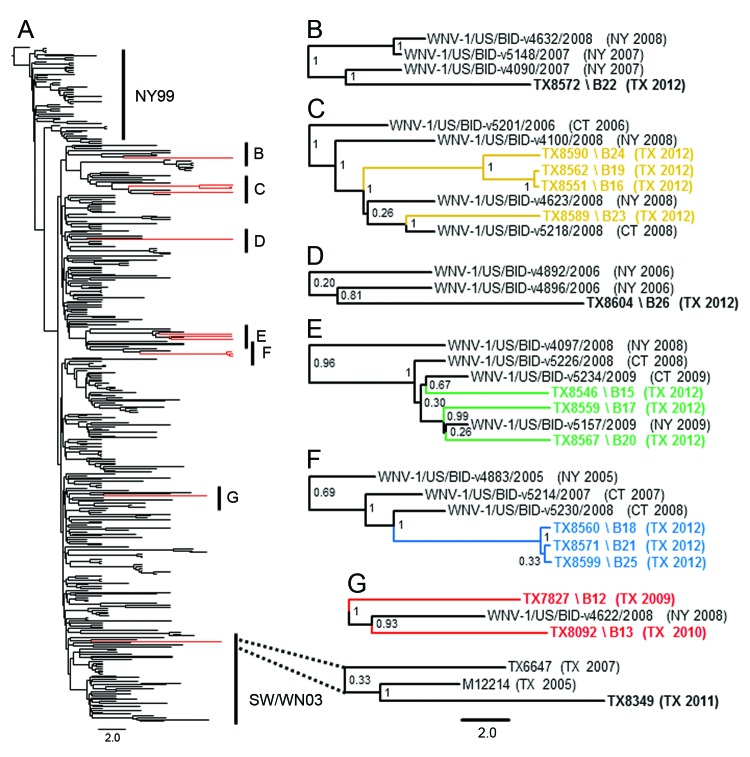
Evolution of West Nile virus (WNV) in North America, 1999–2012. A) Bayesian coalescent tree of all published North American WNV isolates. The NY99 (NY99) and southwestern (SW/WN03) genotypes flank the North American (NA/WN02) genotype containing inferred monophyletic lineages B–G of the novel 2010–2012 Harris County, Texas, WNV isolates. Red indicates WNV isolates sequenced in this study. Isolates sequenced in this study are indicated in **boldface**. B) TX8572 2012 Harris County isolate. D) TX8604 2012 Harris County isolate. C and E–G) Proposed monophyletic groups 7–10 described in Figure 1: C) group 10 (yellow); E) group 9 (green); F) group 8 (blue); G) group 7 (red). Dotted lines indicate distribution of the 2011 TX8349 isolate in the SW/WN03 genotype. Posterior probabilities (range 0.00–1.00) are indicated along branches to provide statistical support for inferred topologies. Scale bars indicate divergence time in years.

Inclusion of the fourteen 2010–2012 Harris County isolates in an additional phylogenetic analysis with 358 published North American WNV isolates enabled us to evaluate the potential influence of active WNV transmission in North America on the recent WNV evolution dynamics observed in Harris County. NJ, ML, and Bayesian relaxed clock methods produced consistent overall tree topologies with retention of the published NY99, NA/WN02 (*12*), and SW/WN03 (*14*) genotypes (Figure 3, panel A). Groups 7–10 showed conserved clustering within the NA/WN02 genotype and robust (≥0.90 posterior probabilities) monophyletic support for shared lineage with several 2006–2009 New York and Connecticut isolates (Figure 3, panels B–F). Furthermore, the 2010 TX8092 isolate demonstrated consistent monophyletic clustering within the NA/WN02 genotype with the 2009 TX7827 and additional 2008 New York (WNV-1/US/BID-v4622/2008) isolates (Figure 3, panel G). In contrast, the novel 2011 TX8349 Harris County isolate retained topologic distribution within the SW/WN03 genotype. Outside the identified group 7–10 monophyletic clusters, all inferred basal node topologies exhibited poor statistical support (≤0.70 posterior probabilities) within the NA/WN02 genotype.

Principal support for the identified monophyletic lineages is based on limited nucleotide divergence (<0.65%) and retention of unique substitutions between the 2010 and 2012 Harris County isolates and those from the northeastern United States compared with published isolates from Texas and the southwestern United States. In particular, the group 9 monophyletic lineage (Figure 3, panel E) encodes several conserved substitutions: NS2A-T52I, NS2A-L95F, NS3-S334T, and NS4B-S14I with a single NS2A-H119Y substitution shared between the 2008 New York (WNV-1/US/BID/v4097/2008) outgroup and the 2012 TX8546 isolate. Each isolate also encodes the characteristic E-V159A substitution with the conserved absence of the NS4A-A85T and NS5-K314R substitutions supporting monophyletic distribution and ancestral lineage within the NA/WN02 versus NY99 and SW/WN03 genotypes.

### Harris County Paradigm—Model for WNV Evolution

**Figure 4:**
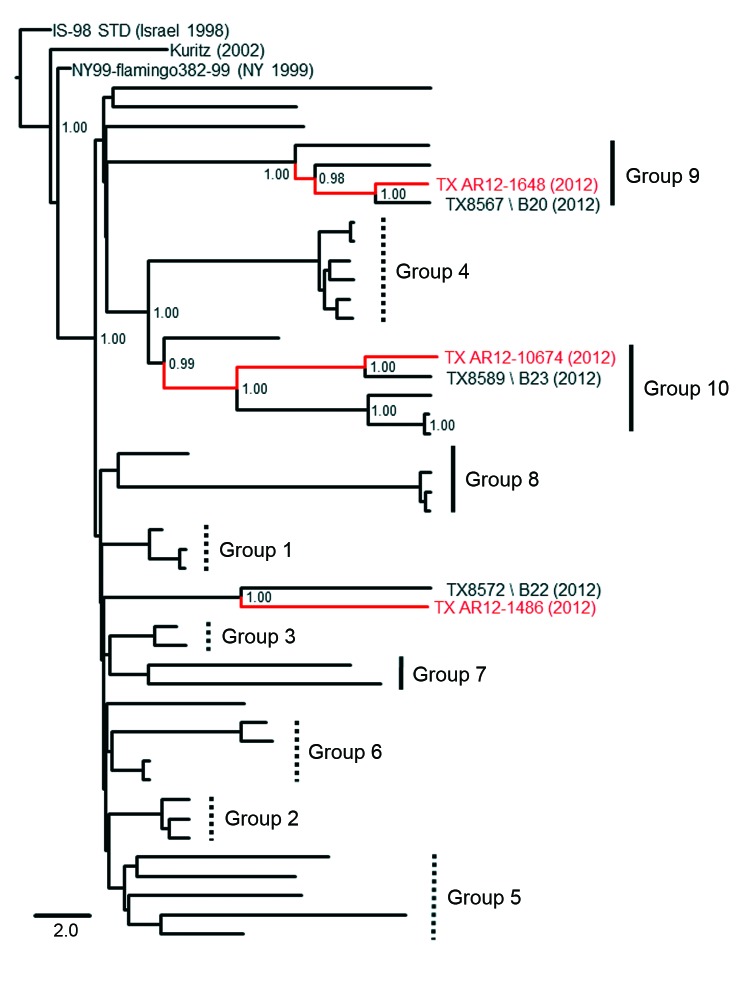
Phylogenetic support for expanded application of the proposed Harris County, Texas, USA, paradigm as a model for West Nile virus (WNV) evolution during the Dallas/Fort Worth, Texas, outbreak, 2012. Bayesian coalescent tree depicts shared monophyletic lineage of the novel Collin County WNV isolates in group 9 (TX AR12-1648) and group 10 (TX AR12-10674) with the TX AR12-1486 Denton County, Texas, isolate clustering with the TX8572 2012 Harris County isolate. Red indicates novel Collin County and Denton County isolates sequenced in this study. Posterior probabilities ≥0.90 are indicated along the branches to provide statistical support for inferred topologies. Scale bar indicates divergence time in years.

To evaluate application of the proposed Harris County paradigm outside the greater Houston metropolitan region, we collected 3 WNV isolates from *Culex* spp. mosquito pools in the recent 2012 Dallas/Fort Worth, Texas WNV outbreak (Table 1) were sequenced and included in a Bayesian phylogenetic analysis with all 42 2002–2012 Harris County isolates. Both Collin County isolates (TX AR12-1648 and TX AR12-10674) exhibited limited nucleotide divergence (≤0.30%) and robust monophyletic clustering (≥0.98 posterior probabilities) with several group 9 and 10 isolates in the Harris County paradigm (Figure 4). In addition, the TX AR12-1468 Denton County isolate demonstrated shared lineage with the nonclustering TX8572 isolate. Overall, our results support potential application of the Harris County paradigm as a relevant model for WNV evolution in Texas as a whole and, possibly, on a national scale.

## Discussion

Surveillance of WNV transmission in Harris County, Texas, provides an excellent model for elucidating the dynamics of endemic and epidemic WNV evolution on a fine geographic scale. The southeastern coastal region of Texas serves as a temporary roosting site on a major flyway for migratory birds in transit between more temperate and tropical regions of the Americas. Consequently, in addition to resident bird populations, this region hosts more avian species (and possible WNV reservoir hosts) than anywhere else in the United States. Prior applications of the proposed Harris County paradigm confirmed emergence of NA/WN02 (*12*) and SW/WN03 (*14*) genotypes from the United States in 2002 and 2003, respectively, as a surrogate model for WNV evolution on a national scale. Our analyses support emergence of 4 novel monophyletic groups of 2010–2012 Harris County isolates (groups 7–10) distinct from group 1–6 phylogenetic clusters identified (Figure 1) (*14*). Furthermore, these isolates exhibit closer ancestral lineage with 2002–2003 Harris County isolates compared with more recent 2007–2009 strains. The single 2011 TX8349 isolate clusters with several 2005–2008 isolates within the group 5 SW/WN03 genotype with conserved expression of the signature NS4A-A85T substitution.

Restriction of conserved substitutions to individual monophyletic groups (Table 2) indicates increased genetic heterogeneity among circulating 2010–2012 populations than with earlier 2002–2009 Harris County WNV populations. Geographic reconstruction of the 42 WNV isolates on a map of Harris County based on mosquito control operational areas (Figure 2) supports a heterogeneous transmission model with limited correlation between fine-scale geographic dispersion and sequence divergence over time (*9*,*10*). However, regional phylogenetic foci among group 2, 4, and 5 mosquito pools and group 8 avian isolates supports potential homogeneous and trans-seasonal WNV transmission in localized vector populations as documented in other studies (*11*,*13*,*33*).

Before the 2010–2012 phylogenetic analyses, a relative stasis in WNV evolution had been observed in Harris County (*16*) and the northeastern United States (*11*,*13*,*17*,*33*) after confirmed emergence of the NA/WN02 and SW/WN03 genotypes. Fine-scale geographic phylogenetic analyses in suburban Chicago and Illinois have identified maintenance and active evolution of heterogeneous WNV populations in local mosquito and avian vectors from the initial introduction into the United States in 1999 until 2008 but not more recently (*9*,*10*,*18*). Applied genomic and phylogenetic comparisons highlight the major divergence of the fourteen 2010–2012 Harris County isolates from historical dynamics of local WNV evolution and emergence of 3 distinct monophyletic lineages (groups 8–10) in 2012 (Figure 1). However, the 2010 and 2012 Harris County isolates exhibit major sequence divergence (>0.42%) relative to group 1–6 isolates despite shared geographic distribution and environmental conditions. In addition, co-circulation of these novel genetic signatures was confirmed in 3 *Culex* spp. mosquito pool isolates obtained from the 2012 WNV outbreak in the greater Dallas/Fort Worth region. On the basis of these observations, introduction of a novel or existing strain from the United States into the circulating greater Houston WNV populations, and possibly Texas as a whole, since 2010 offers an alternative explanation for these divergent genetic signatures. However, our results do not exclude possible emergence of related existent viral populations as dominant regional strains in the recent epidemic WNV transmission season.

To test these hypotheses, we included fourteen 2010–2012 Harris County isolates in a comprehensive Bayesian phylogenetic analysis with all 358 published North American WNV isolates. Shared lineage of group 7–10 isolates with several 2006–2009 strains form the northeastern United States within the NA/WN02 genotype was identified with robust monophyletic support (≥0.90 posterior probabilities) (Figure 3, panels B–G). Principal evidence for these monophyletic lineages is highlighted on the basis of limited divergence (≤0.65%) and conserved expression of unique substitutions relative to observed genetic diversity (0.8%–1.0%) between published group 1–6 strains from the southwestern United States (*14*).

Our results support inferred lineage of 2010 and 2012 Harris County isolates from ancestral strains in the northeastern United States, consistent with a single or multiple introduction event(s) during 2010–2012 in the greater Houston region. However, poor statistical confidence (≤0.70 posterior probabilities) for all inferred basal node topologies within the NA/WN02 genotype limits direct comparison of independent evolution between Harris County isolates in different monophyletic lineages. Furthermore, consistent phylogenetic grouping of the 2011 TX8349 isolate within the SW/WN03 genotype indicates co-circulation of this genotype in Harris County until the 2012 transmission season. However, limited clustering of sequenced 2010–2012 Harris County isolates within the SW/WN03 genotype may be an artifact of inherent bias in sample collection.

Sampling bias is a recognized constraint in phylogenetic and paired geospatial analyses. Unfortunately, the predominance of WNV surveillance remains restricted to limited regional foci. Of the 372 full-length WNV isolates in our analyses, 31.5% (n = 117) were collected in Connecticut, 22.0% (n = 82) in New York, 16.4% (n = 61) in Texas, and 8.1% (n = 30) in Illinois during 1999–2012. In contrast, a single isolate has been characterized for each of the midwestern/central states of Michigan, North Dakota, and South Dakota, all of which reported a major increase in the number of clinical WNV cases (n≥89) in the 2012 epidemic season not seen since 2007 (*15*). Increased WNV surveillance in mosquito and avian vector populations across the entire United States is needed to provide critical, unbiased insight into the underlying dynamics of WNV evolution and transmission in US host populations.

The Harris County Public Health and Environmental Services Mosquito Control Division, which was founded in response to a St. Louis encephalitis virus outbreak in 1964, provides a model infrastructure for WNV surveillance in resident mosquito and avian populations in the greater Houston region (*8*). Uninterrupted collection and processing of WNV-positive bird and mosquito pools since the 2002 introduction of WNV into Harris County has provided a conduit for the scientific investigation of real-time disease outbreaks with direct translation of findings towards optimized vector-borne disease control and prevention. Incorporation of this paradigm in public health directives across the United States would provide a proactive approach towards detection and response to clinical outbreak scenarios of endemic and exotic pathogens in the United States.

In conclusion, retrospective analysis of WNV evolution in Harris County over the past decade indicates a recent shift in the genetic and phylogenetic signature of circulating WNV populations, designated the Harris County paradigm. Further evidence supports introduction of a strain into Texas from the northeastern United States since 2010. Continued WNV surveillance is needed to confirm the effect of this genetic shift in the transmission dynamics and incidence of clinical WNV disease in the greater Houston and surrounding US regions.

Technical Appendix Amino acid substitutions in West Nile virus isolates from Harris County, Texas, USA, 2002–2012.
